# *QuickStats:* Percentage* of Residential Care Communities,^†^ by U.S. Census Region^§^ — National Study of Long-Term Care Providers, 2012–2016

**DOI:** 10.15585/mmwr.mm6743a7

**Published:** 2018-11-02

**Figure:**
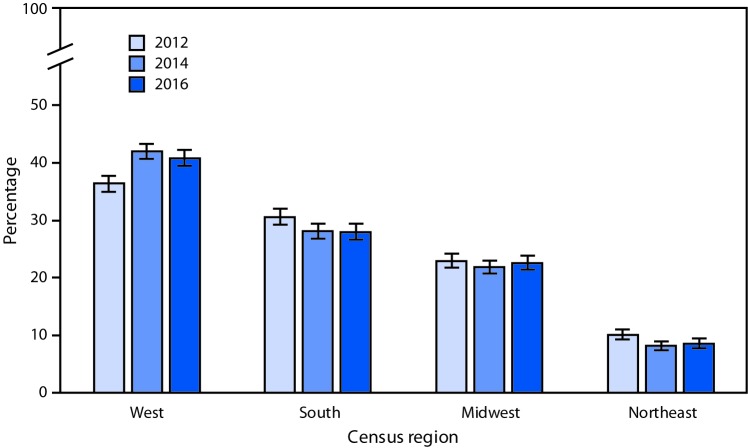
During 2012–2016, the percentage of residential care communities located in the West increased from 36.4% to 40.8%. Throughout the period, a higher percentage of residential care communities were located in the West compared with other regions. The percentage of residential care communities declined from 30.6% in 2012 to 28% in 2016 in the South and from 10.1% to 8.6% in the Northeast. In the Midwest, the percentage was 22.9% in 2012 and 22.6% in 2016.

**Source:** National Study of Long-Term Care Providers, 2012–2016 data. https://www.cdc.gov/nchs/nsltcp/index.htm.

